# Integrative review of factors related to the nursing diagnosis nausea during antineoplastic chemotherapy**^1^**

**DOI:** 10.1590/1518-8345.1176.2812

**Published:** 2016-10-10

**Authors:** Aline Maria Bonini Moysés, Lais Corsino Durant, Ana Maria de Almeida, Thais de Oliveira Gozzo

**Affiliations:** 2 MSc, RN, Hospital das Clínicas, Faculdade de Medicina de Ribeirão Preto, Universidade de São Paulo, Ribeirão Preto, SP, Brazil.; 3 Master's student, Escola de Enfermagem de Ribeirão Preto, Universidade de São Paulo, PAHO/WHO Collaborating Centre for Nursing Research Development, Ribeirão Preto, SP, Brazil.; 4 PHD, Associate Professor, Escola de Enfermagem de Ribeirão Preto, Universidade de São Paulo, PAHO/WHO Collaborating Centre for Nursing Research Development, Ribeirão Preto, SP, Brazil.; 5 PHD, Professor, Escola de Enfermagem de Ribeirão Preto, Universidade de São Paulo, PAHO/WHO Collaborating Centre for Nursing Research Development, Ribeirão Preto, SP, Brazil.

**Keywords:** Nausea, Chemotherapy, Neoplasms, Nursing Diagnosis, Review Literature as Topic

## Abstract

**Objective:**

to identify factors related to the nursing diagnosis nausea among cancer patients undergoing chemotherapy.

**Method:**

integrative review conducted in four electronic databases (PUBMED, EMBASE, CINAHL and LILACS) using the key words: neoplasia, antineoplastic agents and nausea.

**Results:**

only 30 out of 1,258 papers identified met the inclusion criteria. The most frequent related factors were: being younger than 50 years old, motion sickness, being a woman, emetogenic potential of the chemotherapy, anxiety, conditioned stimulus, and expecting nausea after treatment.

**Conclusion:**

this review's findings, coupled with the incidence of nausea among cancer patients undergoing chemotherapy, reveal an important difference between evidence found and that used by NANDA International, Inc. Even though it provides an appropriate definition of related factors, it does not mention chemotherapy, despite the various studies addressing the topic using different designs and presenting various objectives and outcomes.

## Introduction

The incidence of nausea during chemotherapy ranges from 37 to 70%^1^. This incidence is mainly related to the emetogenic potential of chemotherapeutic drugs associated with the individual variations of each patient. Being aware of a drug's emetogenic potential and the characteristics of this adverse event in terms of peak and interval of occurrence is key and essential for the practice of oncologic nursing^2^. 

The ability to control vomiting during chemotherapy has improved considerably in recent years, essentially with regard to drug management, with the development and use of new and modern antiemetic drugs. Controlling nausea, however, seems to be a challenge^3^, and yet the healthcare staff and patients themselves have neglected this symptom.

Some of the likely reasons chemotherapy-induced nausea is difficult to control include: the subjectivity of the symptom, lack of focus when assessing it, lack of validated instruments, limited understanding of physiopathology, inefficient record of this event on the part of patients, and failure on the part of nurses to assess its impact in the lives of patients, especially quality of life^4^.

The impact of nausea on the patients' nutritional status and quality of life become evident few days after chemotherapy. Because in most cases the treatment is administered in an outpatient clinic, this symptom that requires careful assessment is neglected, hindering specific clinical management and nutritional interventions, in addition to the combination of pharmacological and non-pharmacological therapies^5^.

The identification and planning of nursing care concerning the nursing diagnosis (ND) nausea values the work of nurses in the Oncology field and improves the quality of care provided to patients. For this reason, investigating the factors related to this diagnosis is key. Factors related to the ND nausea can favor management and allow the nursing staff to devise a care plan that enables the implementation of efficacious and immediate actions to solve problems^6^. 

Given the previous discussion, this study's objective was to identify the factors related to the nursing diagnosis nausea among cancer patients during chemotherapy through an integrative review.

## Method

An integrative review involves six steps^7^ ^-^ ^8^: identifying the theme and establishing the guiding question or hypothesis, establishing inclusion and exclusion criteria, literature search, deciding on what information will be extracted from the studies selected, assessing the studies included in the review, interpreting results, and synthesizing knowledge. 

This integrative review's aim was to answer the guiding question: *What is the evidence available regarding nausea-related factors among cancer patients undergoing antineoplastic chemotherapy?* For that, a bibliographic search was conducted in four databases: PUBMED (United States National Library of Medicine), LILACS (Latin American and Caribbean Health Sciences Literature), CINAHL (Cumulative Index to Nursing and Allied Health Literature) and EMBASE (Excerpta Medica Database). The search was performed in May 2013, as part of a larger study intended to validate the nursing diagnosis Nausea. No time limit was established for the inclusion of papers.

**Figure 1:**
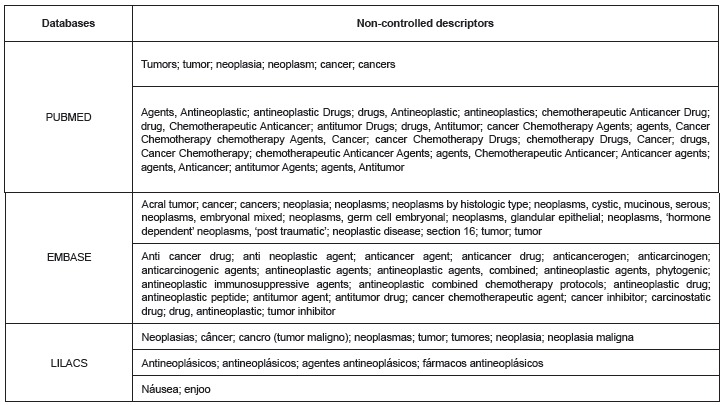
Distribution of databases according to non-controlled descriptors used to search for studies to be included in the integrative review. Ribeirão Preto, SP, Brazil, 2013.

Controlled descriptors used in the search strategies included: *neoplasms, antineoplastic agents* and *nausea*. It is important to note that there were variations, especially with regard to non-controlled descriptors in the different databases, as shown in Figure 1. CINAHL did not present non-controlled descriptors. The Boolean operator AND was used with controlled descriptors and OR was used for non-controlled descriptors; NOT was also used for radiotherapy, surgery, metastasis and chemotherapy.

Criteria used to include papers were: full texts of primary studies addressing nausea in humans undergoing intravenous antineoplastic chemotherapy; written in Portuguese, English or Spanish. Exclusion criteria were: letters, editorials, case studies, pilot studies; papers addressing patients with advanced or metastatic cancer or receiving palliative treatment concomitantly with antineoplastic chemotherapy. 

After the study authors reached a consensus regarding relevant information in each study, data were synthesized. Thus, all papers in the sample were read, aiming to explore the texts and extract data using an instrument^9^ addressing the following items: identification of studies, introduction, objectives, methodological characteristics, results and conclusions. The instrument was completed for all the papers, which were cataloged in ascending order according to year of publication.

All data concerning the characteristics of each study, as well as information regarding nausea-related factors among cancer patients undergoing chemotherapy were grouped in tables and analyzed descriptively. 

The studies' level of evidence was classified according to Melnyk and Fineout-Overholt^10^, following the classification: Level 1 - strong evidence (systematic review or meta analysis); Level 2 - strong evidence (well-designed randomized controlled clinical trials); Level 3 - moderate evidence (non-randomized controlled clinical trials); Level 4 - moderate evidence (case control or cohort studies); Level 5 - weak evidence (systematic reviews, descriptive and qualitative studies); Level 6 - weak evidence (descriptive or qualitative studies); Level 7 - weak evidence (opinion of authorities and/or reports of expert committees). To apply this classification, we first identified the design of each study; only 17 out of the 30 studies presented this information. Two researchers determined the designs of the remaining studies after reading the texts.

## Results

The final sample comprised 30 studies^11^ ^-^ ^40^, 29 of which were indexed in PUBMED and one in EMBASE; 29 were published in English and one in Spanish.

The number of authors ranged from one to seven; most (78.6%) studies were written by up to three authors. Considering the large number of authors, we opted to characterize only the primary author: six papers were authored by psychologists, four by physicians, four by pharmacists, three were written by nurses, and the background of the authors of 11 papers was not identified. 

With regard to the studies' settings, 10 studies were multicenter studies and 16 were conducted at a single center - Oncology day hospitals, General Hospitals or Clinical Research Centers among others; four studies did not report the study setting.

With regard to the country of origin of the primary author, 17 were from the United States of America, three from Spain, two from Greece, one from Sweden, one from Colombia, one from the United Kingdom, one from Austria, one from Thailand, one from Singapore and one from Malaysia.

The periodicals the papers were published in belonged to different health fields: three were from the field of psychology, four from the medical field, two from the field of pharmacy, three journals published papers from the medical and psychology field, and 18 journals were interdisciplinary. Interdisciplinary journals published most (60%) of the papers, including those from the nursing fields.

Because a time limit was not established, the sample included papers published between 1985 and 2012: one was from the 1980s, 10 papers were from the 1990s, 15 were published between 2000 and 2009 and four papers were published from 2010 to 2012.

As shown in Figure 3, the sample was composed of 12 studies with evidence level 6, 14 studies with evidence level 4, one study with evidence level 3, and three studies with evidence level 2.

**Figure 2:**
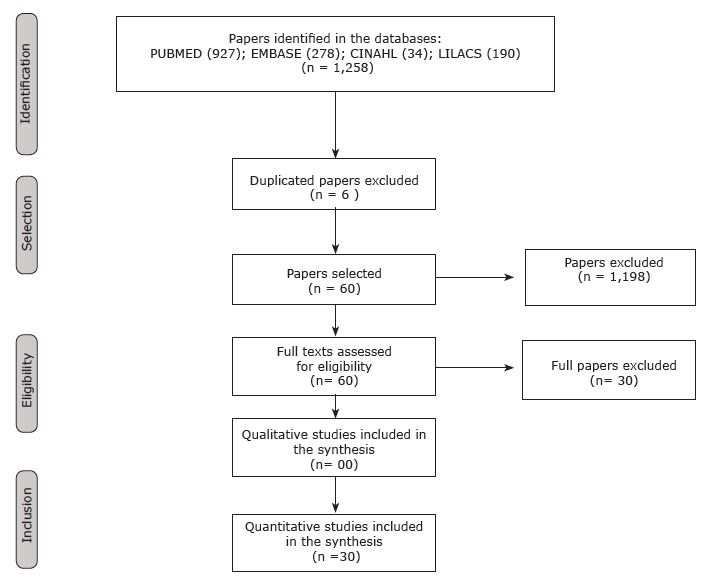
Flowchart of identification, selection, and inclusion of studies.

**Figure 3:**
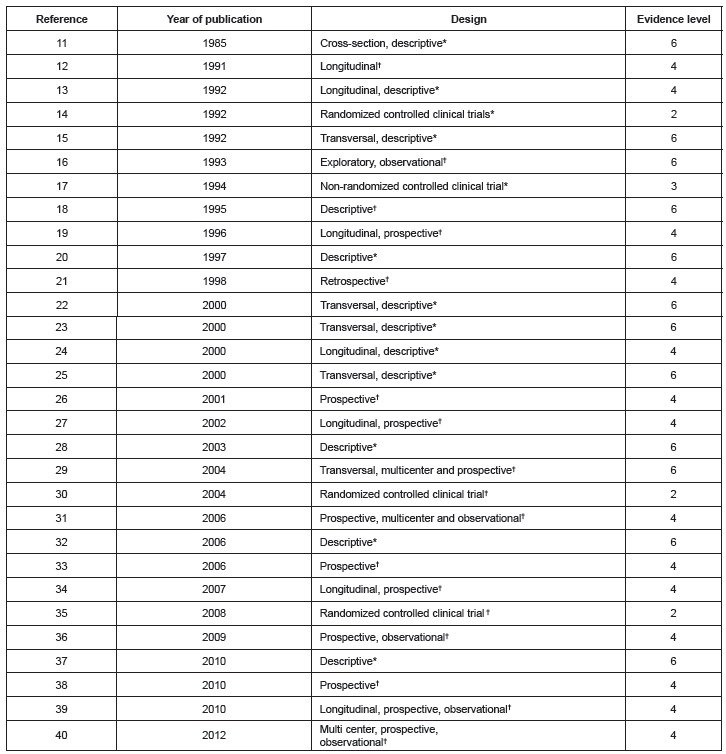
Distribution of studies included in the integrative review according to year of publication, design, and level of evidence. Ribeirão Preto, SP, Brazil, 2013

*Type of study according to this study's authors; † Type of study reported by the papers' authors.

**Figure 4:**
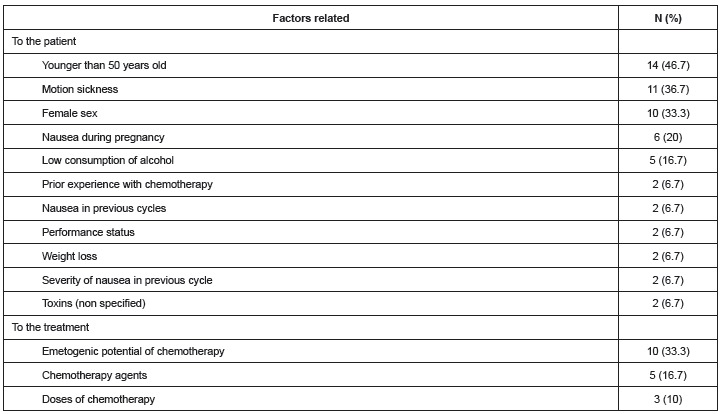
Distribution of absolute (N) and relative (%) frequency of factors related to nausea in the studies included in the integrative review. Ribeirão Preto, SP, Brazil, 2013

A total of 44 related factors were extracted from the studies included in the integrative review: 25 (56.8%) were related to patient, 11 (25%) related to psychological factors, and eight (18.2%) factors were related to treatment. The most frequent were: being younger than 50 years of age, motion sickness, being a woman, anxiety, conditioned stimuli, expecting nausea after treatment, and the emetogenic potential of the chemotherapy. Figure 4 presents the most frequent related factors reported in the studies.

## Discussion

The most frequent factor related to the ND nausea while undergoing chemotherapy was being younger than 50 years old, which was reported by 14 studies^12^ ^-^ ^13^ ^,^ ^20^ ^-^ ^21^ ^,^ ^23^ ^,^ ^26^ ^-^ ^28^ ^,^ ^30^ ^,^ ^32^ ^,^ ^35^ ^-^ ^38^. Young patients more frequently reported acute and late nausea induced by antineoplastic chemotherapy than older patients in all the treatment cycles^41^, and also reported that these symptoms negatively impacted their daily lives^42^. 

One study aimed to assess the predictive power of personal characteristics related to the development of chemotherapy-induced nausea and vomiting (CINV) in a heterogeneous sample of 991 patients with different types of cancer, concluding that the key variables that characterized antecedents for nausea included: not using antiemetic medication according to international guidelines, being young, experiencing nausea before chemotherapy, and inappropriate response to CINV in previous cycle^43^.

Among the factors related to patients, we highlight susceptibility to motion sickness, also known as kinetosis, which was identified in 11 studies^12^ ^,^ ^15^ ^,^ ^20^ ^,^ ^22^ ^-^ ^23^ ^,^ ^26^ ^-^ ^27^ ^,^ ^30^ ^,^ ^35^ ^-^ ^37^.

Motion sickness causes discomfort when movement disturbs the organs responsible for balance. It refers to a combination of autonomic and cognitive signs and symptoms induced when exposed to certain types of movement and may include nausea, vomiting, paleness, cold sweats, hyper salivation, and headache. The control and prevention of these symptoms include pharmacological, behavioral and complementary therapies^44^ ^-^ ^45^.

The Aurélio dictionary emphasizes, in its definition of nausea, that motion sickness is an "unpleasant sensation, well described by familiar expressions, such as having a gag reflex and upset stomach. Associated to feeling nauseated when travelling, especially on water."^46^

A prospective observational study conducted with 213 women with gynecological cancer, which aimed to investigate risk factors for CINV, revealed that being young, motion sickness and the high emetogenic potential of chemotherapy are associated with late nausea^47^.

Another individual factor was the patient's sex. Nausea was more frequently identified among women than among men^20^ ^-^ ^21^ ^,^ ^23^ ^,^ ^27^ ^,^ ^28^ ^,^ ^30^ ^,^ ^35^ ^-^ ^38^. The same was observed with regard to acute nausea, which affected 48% of women compared to 18% of men, as well as for late nausea, which affected 75% of women versus 51% of men^40^.

The incidence of more frequent nausea and vomiting among women may be explained by the frequent use of protocols with higher emetogenic potential and a low consumption of alcohol^48^.

Data also reported by a study investigating risk factors show that female patients are significantly more likely to experience CIVN. Additionally, the following risk factors stand out in the acute phase: being a woman, younger than 55 years of age, and non-habitual consumption of alcohol. In the late phase, only being a woman is a risk factor for CIVN ^(^ ^49^.

The studies under analysis also reported that the emetogenic potential of chemotherapy agents was the most frequent related factor, which was identified in 10 studies^13^ ^-^ ^14^ ^,^ ^17^ ^,^ ^20^ ^,^ ^22^ ^-^ ^23^ ^,^ ^31^ ^,^ ^35^ ^,^ ^38^ ^,^ ^40^.

Some factors that triggered CINV are related to the treatment, including the antiemetic regimen adopted, the specific agent, dose of chemotherapy, route and speed of administration. Short intravenous infusions more frequently induce vomiting than prolonged infusions or oral medications^48^.

Patients treated with highly emetogenic antineoplastic chemotherapy were 5.61 times more likely to experience chemotherapy-induced nausea and vomiting in the first cycle than when treated with moderately emetogenic protocols^41^. The occurrence of anticipatory nausea was also significantly associated with the high emetogenic potential of chemotherapy^50^.

Being a woman and moderate to highly emetogenic chemotherapy stood out among the risk factors that trigger nausea^51^.

With regard to psychological factors, anxiety was the most frequently reported in research^11^ ^,^ ^13^ ^,^ ^20^ ^,^ ^25^ ^-^ ^27^ ^,^ ^34^ ^,^ ^36^ ^-^ ^37^ ^).^ Some anxiety symptoms, such as fear of death, fear for the worse, inability to relax, hot or cold sweats, nervousness, weakness, and numbness were identified as potential indicators of chemotherapy-induced nausea and vomiting^52^. Similarly, the level of pre-treatment anxiety and expectation of nausea and vomiting were strongly associated with the development of severe symptoms^53^. 

A study conducted with 94 women with gynecological cancer, which employed the State-Trait Anxiety Inventory and a self-report questionnaire, reported that being young and anxiety levels are associated with a high risk of experiencing CINV^54^.

The development of anticipatory nausea and vomiting better suits the Pavlovian conditioning model. In this model, a conditioned stimulus, such as the view of a nurse, is paired with an unconditioned stimulus, for instance chemotherapy, which produces an unconditioned response such as nausea. There are no data on the development, clinical progress, or treatment of anticipation adverse events that disagree with this model^55^. Conditioned stimuli were reported in nine studies^11^ ^-^ ^14^ ^,^ ^16^ ^,^ ^19^ ^-^ ^20^ ^,^ ^27^ ^,^ ^34^ ^)^ as being a factor related to chemotherapy-induced nausea.

After experiencing repetitive cycles in which stimuli are paired with subsequent nausea, cycles acquire the ability to trigger nausea and vomiting even before chemotherapy is administered^56^.

Expecting nausea after the treatment was reported in seven studies^13^ ^,^ ^23^ ^,^ ^26^ ^-^ ^27^ ^,^ ^30^ ^,^ ^33^ ^,^ ^35^ included in the integrative review. Patients classified as having a high expectation of experiencing nausea presented the highest mean of occurrence of nausea when compared to those classified as having low expectation^57^. 

The need to investigate nausea-related factors becomes evident, but such an investigation as well as the planning of nursing care should be individualized because factors related to each patient and the emetogenic potential of the regimen should be taken into account.

Note that the best method to avoid or decrease the intensity of nausea and vomiting is to properly prevent them from the first exposure to chemotherapy.

Still in this context, we draw attention to the inexistence of a ND risk for nausea in NANDA-I, which we believe to be relevant to patients who are about to initiate chemotherapy. Devising a way to prevent nausea, planning and implementing actions before the first cycle of chemotherapy is administered should be the focus of nursing care provided to this clientele in order to diminish its negative impact on the patients' nutritional status and quality of life. 

There is a need for nurses to investigate the presence of the nausea-related factors reported in this study. Such an investigation, however, as well as the planning of nursing care should be individualized, taking into account the risk factors of each patient in addition to the emetogenic potential of each treatment regimen.

## Conclusion

The most frequent factors related to the ND nausea presented by patients undergoing antineoplastic chemotherapy were: being young, being a woman, motion sickness, the emetogenic potential of chemotherapy, anxiety, conditioned stimulus, and expectation that nausea will be experienced after the treatment. Of these, only motion sickness and anxiety are factors related to the ND Nausea containing in the North American Nursing Diagnosis Association-International (NANDA-I Inc), which shows an important difference between evidence found and that used by NANDA-I Inc.

This analysis shows that, although the current definition of nausea presented by NANDA-I Inc. is appropriate to patients undergoing chemotherapy, this definition does not include among its related factors "*chemotherapy*" or *"antineoplastic chemotherapy"*, despite the various studies addressing this topic, using different designs and presenting different objectives and outcomes 

Given this review's findings and the incidence of nausea among cancer patients undergoing chemotherapy, it is crucial to include the term "*chemotherapy*" or "*antineoplastic chemotherapy*" among the factors related to the ND Nausea presented by NANDA International, Inc.
